# Review on NMR spectroscopic data and recent analytical methods of aristolochic acids and derivatives in *Aristolochia* herbs

**DOI:** 10.1007/s13659-025-00506-x

**Published:** 2025-06-24

**Authors:** Phan Minh Giang, Nguyen Nghia Vu, Vu Thanh Loc, Dong Ngoc Phuc, Ngiem Duc Trong, To Phuong Linh, Tran Thi Thu Thuy

**Affiliations:** 1 Faculty of Chemistry, VNU University of Science, Vietnam National University, Hanoi, 19 Le Thanh Tong Street, Hanoi, Vietnam; 2 Center for Research and Production of Vaccines and Biologicals, Ministry of Health, 135 Lo Duc Street, Hanoi, Vietnam; 3 Institute of Chemistry of Natural Products, Vietnam Academy of Science and Technology, 18 Hoang Quoc Viet Street, Hanoi, Vietnam; 4 Department of Botany, Hanoi University of Pharmacy, 13-15 Le Thanh Tong Street, Hanoi, Vietnam; 5 Institute of New Technology, Academy of Military Science and Technology, 17 Hoang Sam Street, Hanoi, Vietnam

**Keywords:** *Aristolochia*, Aristolochiaceae, Aristolochic acid (AA), Denitroaristolochic acid, LC analysis, NMR data

## Abstract

Aristolochic acids (AAs) are an important group of secondary metabolites in the genus *Aristolochia.* The presence of aristolochic acids infers the potency of many *Aristolochia* herbs used for ages in traditional medicine of China, Europe, Central America, India, and some other countries. Although being moderately cytotoxic, intake of AAs is associated with serious health problems, such as nephrotoxicity and carcinogenicity. Analyzing AAs in *Aristolochia* herbs is crucial for regulating their efficacy and toxicity because phytochemistry works have shown the occurrence of AAs in almost all *Aristolochia* herbs studied. Using two-dimensional parameters, chemical shifts and coupling constants, NMR spectroscopy is a modern, accurate, and reliable method in the analysis of secondary metabolites. Comparing experimental spectroscopic data with those of known and related compounds helps simplify the structural identification of secondary metabolites. The compilation of an NMR database of AAs from scattered sources would also be useful in NMR-based metabolomics. The present review provides updated information on sources and NMR spectroscopic data of 54 aristolochic acid derivatives, including AAs and their methyl esters, denitroaristolochic acids and their derivatives, and sesqui- and diterpene esters of AAs. The report also covers the newest development of analytical and preparative methods used in separation, identification, and quantification of AAs in *Aristolochia* herbal samples.

**Graphical Abstract:**

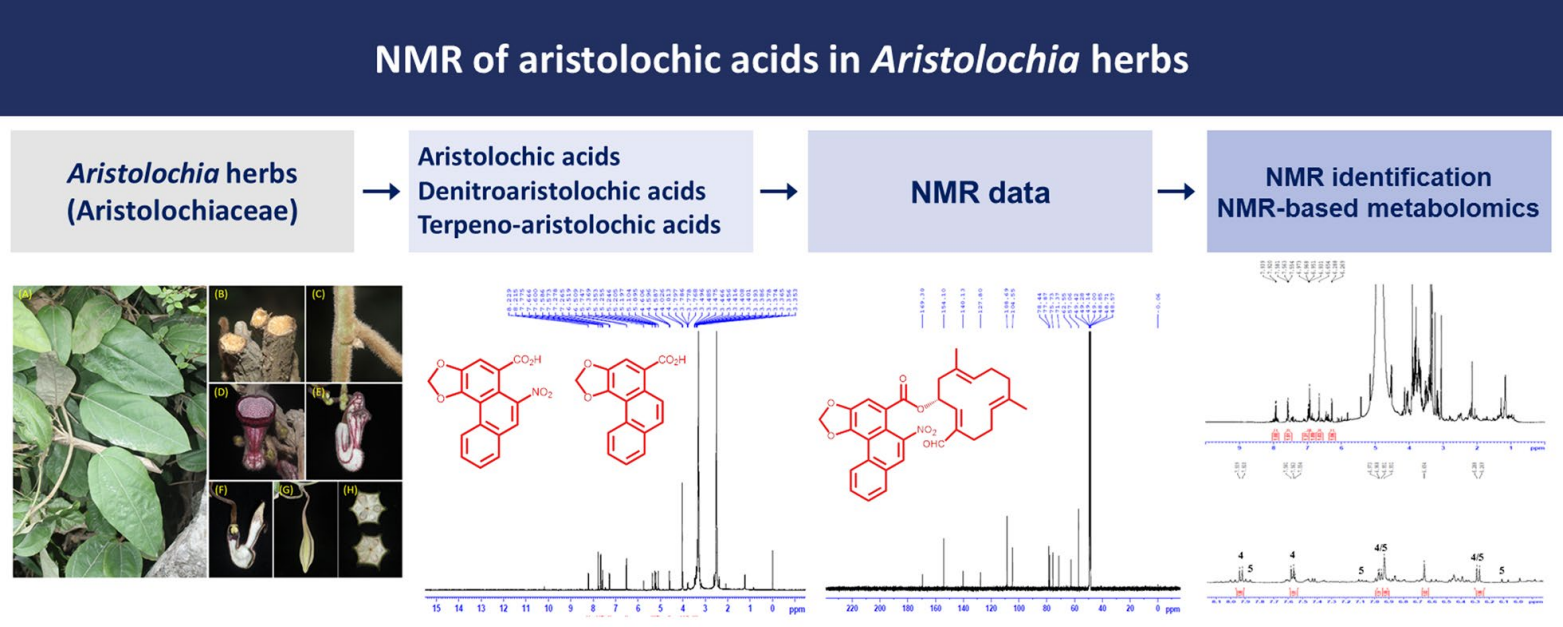

## Introduction

The genus *Aristolochia* comprises more than 500 plant species that grow in wide areas, from tropics to temperate zones. For a long time, members of the genus have been in records for medicinal use in Europe, Asia, Africa, and Central America [1, 2]. Certain *Aristolochia* species have a long tradition to be used in China as popular medicaments in obstetrics, in the treatment of festering wounds, asthma, inflammation, and tumors, and as anodynes, expectorants, and tonics [1, 3–11]. A thorough review published in 2004 lists traditional/folkore medicinal uses of 35 *Aristolochia* species [12]. More than sixty species of *Aristolochia* species have been the subjects of phytochemical and pharmacological studies over the past 70 years [12]. Various types of compounds have been reported from the isolation works, and aristolochic acids (AAs), aristolactams, alkaloids (aporphines, tetrahydroisoquinolines, benzylisoquinolines, and bisbenzylisoquinolines), terpenoids, lignoids, flavonoids (flavones, dihydroflavonols, isoflavonols, biflavones, chalcone-flavones, and tetraflavonoids), coumarins, and quinones are the most common types of compounds [12, 13].

Aristolochic acids are naturally occurring nitrophenanthrenic compounds in *Aristolochia* species of the family Aristolochiaceae, with aristolochic acids I and II usually being the most abundant. In 2014, the chemistry, biosynthesis, and pharmacology of AAs were reviewed [14]. The only source of these substances is the plant itself, and there is currently no large-scale method to efficiently synthesize AAs [15]. The interest in AAs is linked with their possible development into immunostimulants and anticancer agents [16]. Aristolochic acids have been experimentally and clinically demonstrated to be one of the most bioactive constituents of *Aristolochia* herbs, however, the toxicity of these compounds must be understood. Aristolochic acids I and II are the main risk factors for nephropathy and mutagenicity during chronic use of *Aristolochia* herbs for the treatment of rheumatism, diuretics, and analgesics [17]. Intracellular depletion of GSH by aristolochic acid I and long metabolism of aristolochic acids into active intermediates during the detoxification process are the postulated mechanisms for the toxicity [14, 18–20]. In vivo, AAs contribute to the formation of adducts with DNA that result in DNA mutations, leading to the promotion of cancer [14, 20, 21]. In addition, they are the precursors to be metabolized to toxic aristolactams, which may be responsible for the secondary toxicity of *Aristolochia* herbs. Aristolochic acids are also the hypothetical precursors of naturally occurring tariacuripyrones (5-nitro-benzo[h]chromen-2-ones) from *A. brevipes* [22, 23] and aristchamics A and B from *A. championii* [24], whose biological activities and toxicity are not fully understood. Despite being warned on the risks associated with herbal products containing aristolochic acids by reputable official organizations such as the FDA (USA Food and Drug Administration), the WHO International Agency for Research on Cancer, and the Medicines and Healthcare Products Regulatory Agency (MHRA), many websites continue to recommend *Aristolochia* herbal products [25]. Before new regulations to control the safety of these health botanical products may be implemented, more research is needed to ascertain the presence and amounts of AAs in *Aristolochia* plants and to investigate the structure–activity/toxicity relationship between structures of AAs and their curative as well as toxic effects. Several studies pointed out the nitro group as a structural requirement for cytotoxicity of AAs, and the presence or absence of methoxy or hydroxyl groups may mediate cytotoxicicty [14, 26]. It is consistent with the emphasized role of the nitro group in metabolic conversion into harmful intermediates. Moreover, the toxicity rises with the increased number of methoxy groups [14].

Most of the reviews on NMR data of AAs were published prior to 1990. In 1982, 1984, and 1989, the ^1^H-NMR spectroscopic data of AA Ia, AA I (AA A, aristolochic acid), and AA D (AA IVa) [27] and ^13^C-NMR spectroscopic data of some natural (AA I, AA Ia, AA II, AA III, AA IIIa, AA IV, AA IVa, AA Va, aristolic acid and its methyl ester) and synthetic phenanthrene derivatives have been reviewed [27–29]. The ^13^C-NMR data of *ent*-kauranyl aristlochates, aristolin, aristolin I from *A. elegans*, and aristolin II from *A. pubescens* were reported [13]. Recently, more AA derivatives have been isolated and assigned with ^1^H- and ^13^C-NMR techniques, but their spectroscopic data are scattered in the literature. The application of NMR is still valid in the identification of AAs in *Aristolochia* and other plant species, either by isolation or by using NMR-based metabolomics techniques [30]. The aim of the present review is to compile an up-to-date list of NMR spectroscopic data of AAs and their derivatives. In addition, published LC and NMR analytical approaches in the analysis of AAs have been reviewed from the current literature.

## Occurrence of aristolochic acids and their derivatives

According to their structural features, compounds discussed in this review form three characteristic groups: AA derivatives, denitroaristolochic acid derivatives, and sesqui- and diterpene esters of AAs.

### Aristolochic acids, their sodium salts, and their methyl esters

**Fig. 1:**
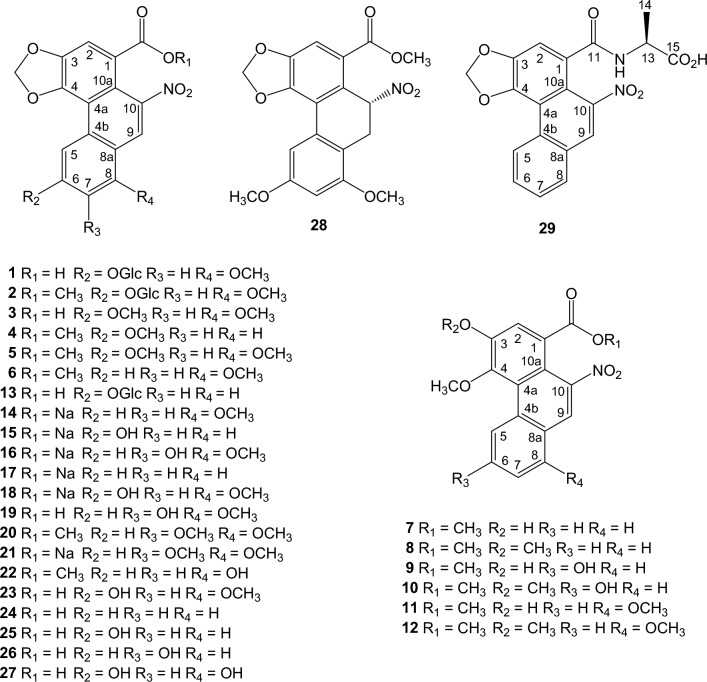
Structures of aristolochic acid derivatives

Aristolochic acid derivatives isolated from *Aristolochia* species are listed in chronological order (Fig. 1). Aristoloside (**1**) was isolated from the stems of *A. manshuriensis* [31] and its semisynthetic methyl ester (**2**), AA III, AA IV (**3**), and AA III methyl ester (**4**) from the root of *A. longa* [32] and from the root and stems of *A. cucurbitifolia* [33]; AA IV methyl ester (**5**) was synthesized from **3** using diazomethane in excess [32], methyl aristolochate (**6**) from the root of *A. indica* [34]*,* 3-hydroxy-4-methoxy-10-nitrophenanthrene-1-carboxylic acid methyl ester (methyl ester of 3-hydroxy-4-methoxy equivalent of AA II) (**7**) from the stems of *A. liukiuensis* (*syn. A. kaempferi*) [35] and the root of *A. auricularia* [16], ariskanins A-E (**8**-**12**) from the root and stems of *A. kankauensis* [36]*,* AA IIIa 6-*O*-*β*-D-glucoside (**13**) from the root of *A. cinnabarina* [37], sodium aristolochate-I (**14**), sodium aristolochate-C (sodium aristolochate IIIa) (**15**), and sodium 7-hydroxyaristolochate A (**16**) from the leaves of *A. foveolata* [38], sodium aristolochate I (**14**), sodium aristolochate II (**17**), sodium aristolochate IIIa (**15**), sodium aristolochate IVa (**18**), and AA VIIa (**19**) from the tubercula of *A. pubescens* [39], aristolochic acid-VII methyl ester (**20**) from the fresh leaves of *A. cucurbitifolia* [40], sodium aristolochate (**14**) and sodium aristolochate-VII (**21**) from the root and stems of *A. heterophylla* [41], AA-Ia methyl ester (**22**) from the root and stems of *A. kaempferi* [42], AA-D (AA IVa) (**23**) from the leaves and stems of *A. bracteolata* [43], AAs B, C, F, and G (**24**-**27**) from the root of *A. fangchi* [17], aristchamic A (**28**) from the rhizomes of *A. championii* [24], AA II (**24**) and AA VIIa (**19**) from the roots of *A. contorta* [18], and AA II alanine amide (**29**) from the whole plant of *A. maurorum* [44].

The substitution of aromatic carbons from C-5 to C-8 of the C-ring of the phenanthrene nucleus may greatly influence the cytotoxicity of AAs. The most cytotoxic is aristolochic acid I with an 8-OMe substituent, followed by aristolochic acid II (unsubstituted), whereas aristolochic acid III (6-OMe) is nontoxic [19]. The cytotoxic potency of AA I is significantly reduced when the 9-hydroxy group is introduced [26]. A reducing trend of cytotoxicity is also observed with 6-hydroxy, 7-hydroxy, and 6,8-dihydroxy substitution [17, 18].

### Denitroaristolochic acids, their sodium salts, and their methyl esters

**Fig. 2:**
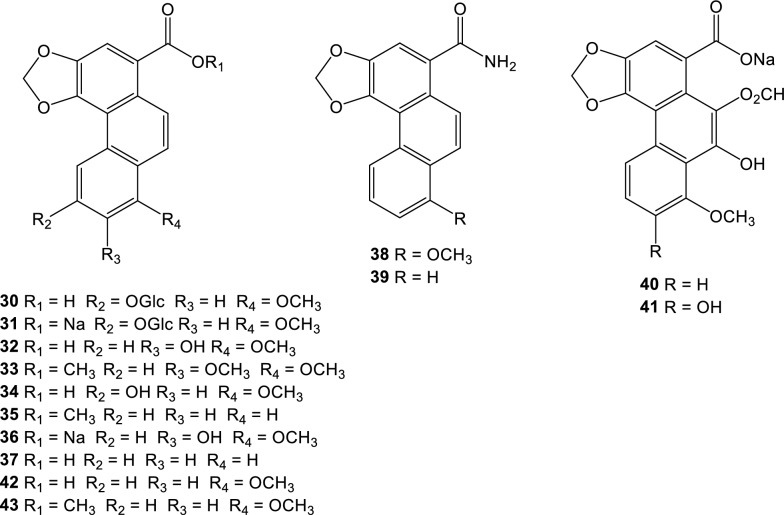
Structures of denitroaristolochic acid derivatives

Aristofolin-A (**30**) were isolated from the flower of *A. kaempferi* [45], sodium aristofolin-A (**31**), aristofolins B, C, and D (**32**-**34**) from the leaves of *A. cucurbitifolia* [46], aristofolin-E (**35**) from the stem and root of *A. kaempferi* [42], and sodium 7-hydroxy-8-methoxyaristolate (**36**) from the root and stem of *A. cucurbitifolia* [33], demethylaristofolin E (**37**) from the stem of *A. manshuriensis* [47], aristolamide (**38**) from the roots of *A. indica* [48] and aristolamide II (**39**) from the stem of *A. manshuriensis* [49], sodium 9-hydroxy-10-formyloxy aristolochate I (**40**) and sodium 7,9-dihydroxy-10-formyloxy aristolochate I (**41**) from the roots of *A. contorta* [18] (Fig. 2). We have updated the ^1^H-NMR spectroscopic data of aristolic acid (**42**) and its methyl ester (**43**), using the experimental data of the corresponding compounds isolated from the Taiwanese butterfly *Pachliopta aristolochiae interpositus*, which exclusively feeds on *A. cucurbitifolia* [50] and the synthetic products [19, 51]. Biogenetically, the nitro group of aristolochic acids can be replaced by hydrogen in a reaction called hydrogenolysis. Experimentally, Priestap *et al.* showed evidence for a conversion of aristolochic acid I into aristolic acid on treatment with cysteine or GSH (glutathione) under physiological conditions [19]. Hydrogenolysis may involve the direct transfer of a hydride ion from the thiol group to eliminate the nitro group at C-10 of aristolochic acid I, leading to the denitro derivative.

Not much information is available about the cytotoxicity of derivatives of aritolic acids. In a few cases when the cytotoxicity of aristolic acid II and aristolic acid is correlated with that of the corresponding AAs, the loss of the nitro group significantly reduces the cytotoxicity of the denitro derivatives of AAs [14].

### Sesqui- and diterpene esters of aristolochic acids

**Fig. 3:**
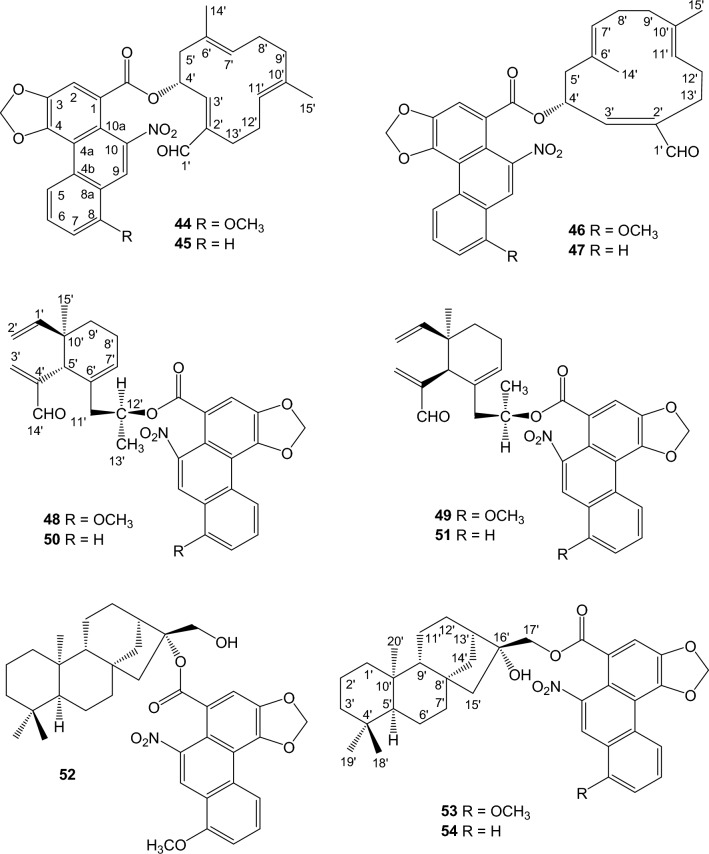
Structures of sesqui- and diterpene esters of aristolochic acids

Hybrid structures of sesquiterpene ester of AAs, including aristoloterpenates I, II, III, and IV (**44-47**) [52] and aristophyllides A, B, C, and D (**48-51**) [53], were isolated from the root and stem of *A. heterophylla*. *ent*-Kaurane diterpene esters of aristolochic acids, aristolin (**52**) and aristoloins I (16*α*-hydroxy-*ent*-17-kauranyl aristolochate I) (**53**) and II (16*α*-hydroxy-*ent*-17-kauranyl aristolochate II) (**54**), were isolated from the root and stem of *A. elegans* [54] and the tubercula of *A. pubescens* [39], respectively (Fig. 3).

## Analytical and preparative separation of aristolochic acids

High-performance liquid chromatography (HPLC) coupled with a photodiode array (PDA) detector is considered one of the most reliable techniques for the analysis of substances with small quantities in biological samples. As the causative agents of nephropathy, analytical HPLC methods have been developed for the qualitative and quantitative analysis of AAs I and II in *Aristolochia* herbs. The analysis of other AAs and aristolactams based on comparison of HPLC retention time often encounters the shortage problem of reference compounds. PDA detectors are often used with the UV detection wavelength set at 250, 254, or 260 nm. Quantification (w/w) was based on calibration curves, which were constructed by plotting area vs. concentration. In general, simple, rapid, and accurate HPLC methods were developed to enable the simultaneous identification of AAs. Aristolochic acids have been found in almost all the *Aristolochia* samples analyzed in these HPLC studies, and the content of aristolochic acid I is usually higher than that of aristolochic acid II. To our surprise, hardly over twenty *Aristolochia* species have been found to contain AAs, making the development and validation of the HPLC analytical methods practically crucial [1].

Hashimoto et al*.* used HPLC with a Waters ODS column and MeOH-1% acetic acid (50:50, v/v) elution to examine AAs I and II in *A. debilis*, *A. fangchi*, and *A. manshuriensis*. The quantitative results showed the levels of AAs I and II in *A. manshuriensis* were higher than the non-toxic effect level established at 0.2 mg/kg [55]. The amounts of AAs I and II in the different parts (root, stem, leaves, fruit) of *A. clematitis* were quantified by Bartha et al. [56]. AAs I and II were found to have the highest contents in the herb’s root. Using a non-acidic methanol–water (60:40) solvent system and an ODS Hypersil C18 column, Li et al. observed a reasonable separation of AAs I and II in *A. fangchi* [57]. Many factors, such as collection region, cultivation methods, and parts of the plants, can affect the contents of AAs. Alali et al. used a HPLC LichroCART^®^ 125–4 column and a MeOH-1% acetic acid (60:40, v/v) solvent system to identify AAs I and II in root, stem, and leaves of *A. maurorum* [58]. The analytical samples were extracted in these experiments using a mixture of 80% MeOH and 20% formic acid in water. Because the parent substances contained both a carboxyl group and a nitro group, the acidic condition was considered favorable to extract AAs from plant materials. Root was found to be the main storage of the two AAs during the flowering stage. Using a C_18_ HPLC method with a Zobrax SB-C_18_ clumn, acetonitrile and 3.7 mM phosphoric acid buffer gradient elution, Zhang et al. successfully separated five AAs (aristolochic acid I, II, IVa, Va, and 9-hydroxyaristolochic acid-1) and two aristolactams (aristolactams I and 2) from the fruit of *A. contorta* (traditional drug name in the *Pharmacopoiea of the People’s Republic of China*: Madouling), root of *A. contorta* (Bei-madouling-gen), herbs of *A. contorta* and *A. debilis* (Tianxianteng), root of *A. debilis* (Quingmuxiang), stem of *A. manshuriensis* (Guanmutong), and root of *A. fangchi* (Guangfangji) [59]. After examining 60 samples, only AAs I and II were detected in the root and herbs of *A. debilis*, while AAs I, II, and IVa were identified in the root of *A. fangchi* and the stem of *A. manshuriensis*. All seven compounds were found in the fruit and herbs of *A. contorta*, with the exception of aristolactam-II, which was absent from the herbs of *A. contorta*. Furthermore, the findings demonstrated that the majority of *Aristolochia* herbs in China and Japan had a significantly higher amount of aristolochic acid I than aristolochic acid II. Using an HPLC method with a Waters C-18 HPLC column, MeOH-1% acetic acid (60:40, v/v), and UV detection at 250 nm, Abdelgadir et al*.* quantified the amounts of AAs I and II in the whole plant of *A. bracteata* collected in Sudan as 12.98 g/kg and 49.03 g/kg, respectively [60]. This is the first report on the higher amount of aristochic acid II than that of aristolochic acid I; this could be due to geographic or biological diversity of the *Aristolochia* species studied [60]. Using an InertSustain C18 column and an isocratic solvent mixture of 0.1% acetic acid in the ratio acetonitrile–water (50:50), Araya et al*.* analyzed AAs I and II in the leaves of *A. chilensis* [61]. To confirm the identification of AAs, each HPLC signal was collected and directly injected into an Orbitrap mass detector; the resulting mass spectra were compared with those of the commercial samples. Rodríguez et al*.* used ethyl acetate to extract AAs from leaves and stems of *A. sprucei*. They used a Supelco™ LC18 column with a photodiode array detector monitored at 254 nm and a solvent gradient of 10–66% MeOH in water for the first 32 min, followed by 66–10% MeOH for the next 32–35 min, and 100% MeOH for the time left, to analyze the crude extracts [62]. Aristolochic acid I was identified in the stem extract, but not in the leaf extract of *A. sprucei*. The chromatographic condition showed the distinct separation of aristolochic acid I from the other compounds in the extracts.

Metabolomics techniques based on LC–ESI–MS and ^1^H-NMR spectroscopy are also used to ascertain the variation of AAs and their derivatives. Michl et al*.* were able to screen aristolochic acid analogues in 43 medicinally used *Aristolochia* species using this method [30]. Metabolomics data processing involves AMIX software for the ^1^H-NMR spectra in the range 0.1–10 ppm and Mzmine 2 for peak extraction, chromatogram deconvolution, and peak alignment in the HR LC–MS data. While most of the compounds were tentatively assigned based on LC–MS accurate mass, retention times, UV maxima, MS/MS fragmentation ions, and ^1^H-NMR data set some analogues were identified by comparison with known reference standards. Consequently, a AAs I and II were found to be the most prevalent members, and that carbohydrates and fatty acids are the primary chemical markers that differentiate *Aristolochia* samples. Based on MS data libraries of Traditional Medicine Zhang et al*.* developed an UP (Ultra-Performance)LC-QTOF-MS/MS method using a Waters Acquity UPLC-BEH C_18_ column, a gradient elution of 0.1% formic acid aqueous solution-acetonitrile, and ESI–MS detection with a full scale mode ranged *m/z* 50–1500 [62]. Compounds were identified in three species of *Aristolochia* herbs, including *A. mollissima*, *A. debilis*, and *A. cinnabaria*, by comparing chromatographic and MS characteristics with those of four aristolochic acid standards and the published literature. To explain the qualitative and quantitative variations in the three *Aristolochia* herbs from different origins, the authors employed 19 differential markers of AAs and aristolatams from *A. mollissima*, 16 differential markers from *A. debilis*, and 22 differential markers from *A. cinnabaria*. Compared to the other species of *A. mollissima* and *A. debilis*, *A. cinnabaria* had much higher levels of AAs.

Capillary electrophoresis (CE) offers many advantages, such as high speed, high efficiency, ultra-small sample volume, and low consumption of solvent [63]. Coupling electrochemical detection (ED) with CE can provide high separation efficiency and good sensitivity. Zhou et al*.* detected AAs I and II in the fruit, stem, and root of *A. debilis* using a 33 μm carbon fiber electrode (CFE) as the working electrode for the [64]. An aqueous buffer with 2.0 × 10^–2^ mol L^−1^ phosphate buffer solution (PBS) (pH 10.0) was used to accomplish the separation. In accordance with the results of many previous analytical reports, the contents of AAs in the root are higher than those in the fruit, but abnormally, they were not detected in the stem.

The recent emphasis on qualitative and quantitative investigation of AAs in the original plants or formulations was highlighted by Zhang et al. [65]. The works require high-purity reference compounds. When using preparative liquid chromatography, the required purity of the target compounds is achieved if the distance between zones of compounds under separation increases. Using Waters Auto-purification Factory with an oligo (ethylene glycol) (OEG) separation column to cluster compounds with similar structures and preparative PDA-HPLC with a C-18 separation column to purify the target compounds the authors develop a method for selective purification of AAs from *Aristolochia* plants. A gradient solvent system with 0.2% formic acid water and methanol and 0.2% formic acid water and acetonitrile were used for fraction separation and compound purification, respectively. The detection wavelength was 260 nm. Aristolochic acids from *A. manshuriensis* were successfully separated and purified using this method. Duan et al*.* used an advanced counter-current chromatography (CCC) technique, called pH-zone-refining CCC (pH-ZRCCC), in preparative isolation of AAs [66]. A large-scale separation technique for organic acids and bases according to their pH in displacement mode, pH-ZRCCC was introduced in 1991. The method uses a retainer base (or acid) to hold the analytes in the column and a displacer acid (base) to elute the analytes in the decreasing order of p*K*_a_ and hydrophorbicity. pH-ZRCCC has been shown to be effective in isolating target compounds from the EtOAc extract from the stem of *A. manshuriensis* with high purity in a single run and is not affected by irreversible adsorption. Petroleum ether-EtOAc–MeOH-water (3:7:3:7, v/v/v/v) was the optimum two-phase solvent system. Triethylamine and trifluoroacetic acid were added to the organic phase. The collection was monitored with a UV detector at 254 nm. The elution order of AAs in the chromatogram (aristolochic acid IIIa > aristolochic acid IVa > aristolochic acid II > aristolochic acid I > aristolic acid II > aristolic acid I) was in accordance with the hydrophorbic properties of AAs and their acidities.

## Structure elucidation of aristolochic acids and derivatives

According to the structural features of isolated AAs from the genus *Aristolochia* they have been grouped into the following groups: i) AAs, their sodium salts, and their methyl esters; ii) denitroaristolochic acids (derivatives of aristolic acids), their sodium salts, and their methyl esters; and iii) sesqui- and diterpene esters of AAs. Tables 1, 2, 3, 4, 5, 6, 7, 8, 9, and 10 list the ^1^H- and ^13^C-NMR spectroscopic data for AAs, denitroaristolochic acids, and terpene esters of AAs. The nonpolar, aprotic CDCl_3_ is used for NMR measurements of less polar methyl esters of AAs, denitroaristolochic acids and terpene esters of AAs, whereas the polar, aprotic DMSO-*d*_6_ is the common NMR solvent for AAs. On a smaller scale, acetone-*d*_6_ is used for methyl esters of AAs and methanol-*d*_4_ is used for polar AA derivatives, such as their glucosides or sodium salts. Due to interactions between NMR solvents and compounds, measured chemical shifts may vary for the same compounds as observed for compound **42** in DMSO-*d*_6_ and acetone-*d*_6_ (Table 8). Since typical structures of AAs are not complex, with most of the signals falling in the downfield region, the resolution of substituted aromatic C-rings from H-5 to H-8 may benefit from high-field NMR techniques. For example, the ^1^H-NMR data of compound **42** having an 8-methoxy substituent in DMSO-*d*_6_ are almost coincident when measured at 60 MHz [51] and 400 MHz [19]. Most of the time, 2D NMR techniques such as HSQC (or HMQC), HMBC, and COSY were used to assign the proton and carbon-13 signals.

**Table 1:** Aristolochic acids and derivatives from *Aristolochia* species

Plant	Plant material	Compound	References
*A. manshuriensis*	Stems	Aristoloside (**1**)	[31]
*A. longa* *A. cucurbitifolia*	Root Root, stems	Aristolochic acid IV (**3**) Aristolochic acid III methyl ester (**4**)	[32] [33]
*A. indica*	Root	Methyl aristolochate (**6**)	[34]
*A. liukiuensis* *A. auricularia*	Stems Root	3-Hydroxy-4-methoxy-10-nitro- phenanthrene-1-carboxylic acid methyl ester (**7**)	[35] [16]
*A. kankauensis*	Root, stems	Ariskanins A-E (**8**–**12**)	[36]
*A. cinnabarina*	Root	Aristolochic acid IIIa 6-*O*-*β*-D-glucoside (**13**)	[37]
*A. foveolata*	Leaves	Sodium aristolochate-I (**14**) Sodium aristolochate-C (**15**) Sodium 7-hydroxyaristolochate A (**16**)	[38] [38] [38]
*A. pubescens*	Tubercula	Sodium aristolochate I (**14**) Sodium aristolochate IIIa (**15**) Sodium aristolochate II (**17**) Sodium aristolochate IVa (**18**) Aristolochic acid VIIa (**19**)	[39] [39] [39] [39] [39]
*A. cucurbitifolia*	Leaves	Aristolochic acid-VII methyl ester (**20**)	[40]
*A. heterophylla*	Root, stems	Sodium aristolochate (**14**) Sodium aristolochate-VII (**21**)	[41] [41]
*A. kaempferi*	Root, stems	Aristolochic acid-Ia methyl ester (**22**)	[42]
*A. bracteolata*	Leaves, stems	Aristolochic acid-D (**23**)	[43]
*A. fangchi*	Root	Aristolochic acids B, C, F, G (**24**–**27**)	[17]
*A. championii*	Rhizomes	Aristchamic A (**28**)	[24]
*A. contorta*	Roots	Aristolochic acid VIIa (**19**) Aristolochic acid II (**24**)	[18] [18]
*A. maurorum*	Whole plant	Aristolochic acid II alanine amide (**29**)	[44]
*A. kaempferi*	Flower	Aristofolin-A (**30**)	[45]
*A. cucurbitifolia*	Leaves	Sodium aristofolin-A (**31**) Aristofolins B, C, and D (**32**–**34**)	[46] [46]
*A. kaempferi*	Root, stems	Aristofolin-E (**35**)	[42]
*A. cucurbitifolia*	Root, stems	Sodium 7-hydroxy-8-methoxyaristolate (**36**)	[33]
*A. manshuriensis*	Stems	Demethylaristofolin E (**37**)	[47]
*A. indica*	Roots	Aristolamide (**38**)	[48]
*A. manshuriensis*	Stems	Aristolamide II (**39**)	[49]
*A. contorta*	Roots	Sodium 9-hydroxy-10-formyloxy aristolochate I (**40**) Sodium 7,9-dihydroxy-10-formyloxy aristolochate I (**41**)	[18] [18]
*A. heterophylla*	Root, stems	Aristoloterpenates I, II, III, IV (**44**–**47**)	[52]
		Aristophyllides A, B, C, D (**48**–**51**)	[53]
*A. elegans*	Root, stems	Aristolin (**52**)	[54]
*A. pubescens*	Tubercula	Aristoloin I (**53**) Aristoloin II (**54**)	[39]

**Table 2:** ^1^H- and ^13^C-NMR characteristic signals of aristolochic acid derivatives (*δ* in ppm,* J* in Hz, DMSO-*d*_6_)

H/C	3,4-Methylenedioxyaristolochic acids		3,4-Methylenedioxyaristolic acid	
	^1^H-NMR	^13^C-NMR	^1^H-NMR	^13^C-NMR
2	*ca.* 7.70–7.80 *s*	*ca.* 111–114	*ca.* 7.70–7.80 *s*	*ca.* 111–114
5	*ca.* 8.20–8.70 *s*	*ca.* 111–130	*ca.* 8.20–8.90 *s*	*ca.* 111–130
9	*ca.* 8.20–8.70 *s*	*ca.* 120–130	*ca.* 8.80 *d* (9.0–10.0)	*ca.* 124–127
10		*ca.* 143–146	*ca.* 7.70 *d* (9.0–10.0)	*ca.* 124–127
O-CH_2_-O	*ca.* 6.40 *s*	*ca.* 102–104	*ca.* 6.40 *s*	*ca.* 102–104
OCH_3_	*ca.* 4.0 *s*	*ca.* 56–61	*ca.* 4.0 *s*	*ca.* 56–57

**Table 3:** ^1^H-NMR spectroscopic data of compounds **1**–**7** (*δ* in ppm,* J* in Hz)

H	**1**^a&^ [31] 90 MHz	**2**^b$^ [31] 200 MHz	**3**^a^ [32] 60 MHz	**4**^c%^ [33] 200 MHz	**5**^c^ [32] 200 MHz	**6**^a^ [34] 60 MHz	**7**^a^ [16] 250 MHz	**7**^d^ [35] 270 MHz
2	7.78 *s*	7.73 *s*	7.70 *s*	7.76 *s*	7.73 *s*	7.80 *s*	7.80 *s*	7.83 *s*
5	8.35 *d* (2.0)	8.45 *dd* (2.1, 0.6)	7.84 *d* (2.0)	8.60 *d* (2.4)	8.13 *d* (2.0)	8.66 *d* (8.1)	9.60 *d* (8.6)	9.60 *br d* (8.7)
6						7.68 *t* (7.7)	7.80 *t* (8.1)	7.82 *m*
7	7.13 *d* (2.0)	6.99 *d* (2.1)	6.78 *d* (2.0)	7.34 *dd* (8.8, 2.4)	6.70 *d* (2.0)	7.28 *d* (8.1)	7.91 *t* (8.1)	7.73 *m*
8				7.90 *d* (8.8)			8.26 *d* (8.1)	8.15 *dd* (6.2, 1.3)
9	8.50 *s*	8.73 *d* (0.6)	8.32 *s*	8.31 *s*	8.74 *s*	8.36 *s*	8.59 *s*	8.41 *s*
O-CH_2_-O	6.49 *s* 6.44 *s*	6.40 *d* (1.2) 6.37 *d* (1.2)	6.39 *s*	6.39 *s*	6.34 *s*	6.43 *s*		
OCH_3_	4.07 *s*	4.06 *s*	3.97 *s* 3.88 *s*	4.02 *s*	3.98 *s* 4.01 *s*	4.05 *s*	3.93 *s*	3.93 s*
CO_2_Me		3.86 *s*		3.87 *s*	3.86 *s*	3.81 *s*	3.73 *s*	3.83 s*

^a^DMSO-*d*_6_

^b^CDCl_3_-CD_3_OD

^c^CDCl_3_

^d^(CD_3_)_2_CO

^e^CD_3_OD

^*^interchangeable

^&^Glc: 5.12 *d* (7.0), 3.2–4.2 *m*

^$^Glc: 5.15 *d* (7.3), 3.98 *dd* (12.0, 2.0), 3.81 *dd* (12.0, 5.0), 3.45–3.70 *m*

^%^OH: 9.47 *s*

**Table 4:** ^1^H-NMR spectroscopic data of compounds **8–16** (*δ* in ppm,* J* in Hz)

H	**8**^c^ [36] unknown	**9**^d&^ [36] unknown	**10**^d$^ [36] unknown	**11**^c%^ [36] unknown	**12**^c^ [36] unknown	**13**^e^^ [37] unknown	**14**^a^ [38] unknown	**15**^a^ [38] unknown	**16**^a*^ [38] unknown
2	7.89 *s*	7.76 *s*	7.89 *s*	7.89 *s*	7.88 *s*	7.68 *s*	7.62 *s*	7.69 *s*	7.70 *s*
5	9.66 *dd* (8.0, 1.8)	9.13 *d* (2.3)	9.21 *d* (2.3)	8.99 *d* (8.8)	9.21 *d* (8.0)	8.88 *d* (2.5)	8.63 *d* (8.2)	8.43 *d* (2.2)	8.65 *d* (9.0)
6	7.81 *td* (8.0, 1.6)			7.74 *t* (8.8)	7.72 *d* (8.0)		7.74 *t* (8.2)		7.48 *d* (9.0)
7	7.71 *td* (8.0, 1.8)	7.34 *dd* (8.5, 2.3)	7.34 *dd* (8.7, 2.3)	7.13 *d* (8.8)	7.11 *d* (8.0)	7.48 *dd* (8.9, 2.5)	7.27 *d* (8.2)	7.21 *dd* (8.4, 2.2)	
8	7.99 *dd* (8.0, 1.6)	8.08 *d* (8.5)	8.07 *d* (8.7)			7.99 *d* (8.9)		7.98 *d* (8.4)	
9	8.34 *s*	8.38 *s*	8.40 *s*	8.87 *s*	8.89 *s*	8.23 *s*	8.30 *s*	8.18 *s*	8.26 *s*
O-CH_2_-O						6.34 *s*, 6.37 *s*	6.35 *s*	6.36 *s*	6.37 *s*
OCH_3_	3.97 *s* 4.10 *s*	3.93 *s*	3.99 *s* 4.10 *s*	3.89 *s* 4.08 *s*	3.94 *s* 4.05 *s* 4.10 *s*		4.03 *s*		3.95 *s*
CO_2_Me	3.88 *s*	3.76 *s*	3.77 *s*	3.85 *s*	3.88 *s*				

^a^DMSO-*d*_6_

^c^CDCl_3_

^d^(CD_3_)_2_CO

^e^CD_3_OD

^&^OH: 9.29 *s*, 9.52 *s*

^$^OH: 9.47 *s*

^%^OH: 6.42 *s*

^^^Glc: 5.14 *d* (6.9), 3.95 *dd* (11.9, 2.0), 3.80 *dd* (11.9, 5.4), 3.58 *m*, 3.50 *d* (11.9)

^*^OH: 10.50* br s*

**Table 5:** ^1^H-NMR spectroscopic data of compounds **19**–**28** (*δ* in ppm,* J* in Hz)

H	**19**^a^^ [18] 500 MHz	**20**^c^ [40] 200 MHz	**21**^a^ [41] 200 MHz	**22**^c^ [42] 200 MHz	**23**^a^ [43] 400 MHz	**24**^a^ [17] 500 MHz	**25**^a&^ [17] 500 MHz	**26**^a#^ [17] 500 MHz	**27**^a$^ [17] 500 MHz	**28**^c^ [24] 500 MHz
2	7.70 *s*	7.70 *s*	7.71 *s*	7.73 *s*	7.69 *s*	7.75 *s*	7.73 *s*	7.72 *s*	7.76 *s*	7.49 *s*
5	8.70 *d* (9.05)	8.85 *d* (9.6)	8.80 *d* (9.5)	8.70 *d* (8.6)	8.06 *d* (2.4)	8.52 *m*	8.42 *s*	8.69 *d* (8.2)	8.13 *d* (2.0)	7.38 *d* (2.3)
6	7.50 *d* (9.05)	7.46 *d* (9.6)	7.74 *d* (9.5)	7.71 *t* (8.6)		7.70 *m*		7.31 *dd* (8.2, 2.2)		
7				7.10 *d* (8.6)	6.84 *d* (2.4)	7.74 *m*	7.23 *d* (8.0)		6.72 *d* (2.0)	6.47 *d* (2.3)
8						7.90 *m*	8.02 *d* (8.0)	7.42 *d* (2.2)		
9	8.40 *s*	8.67 *s*	8.41 *s*	8.82 *s*	8.36 *s*	8.39 *s*	8.42 *s*	8.21 *s*	8.70 *s*	2.81 *dd* (17.6, 5.4) 4.23 *dd* (17.6, 2.4)
10	6.45 *s*									6.67 *d* (2.4, 5.4)
O-CH_2_-O	7.70 *s*	6.37 *s*	6.46 *s*	6.38 *s*	6.40 *s*	6.41 *s*	6.345 *s*	6.43 *s*	6.50 *s*	6.13 *d* (1.4) 6.28 *d* (1.4)
OCH_3_	3.98 *s*	4.04 *s* 4.06 *s*	3.98 *s* 4.00 *s*		3.98 *s*					
CO_2_Me		3.87 *s*		4.06 *s*						
CO_2_H						10.96 *s*	10.88 *s*	10.90 *s*	10.82 *s*	

^a^DMSO-*d*_6_

^c^CDCl_3_

^^^OH: 10.44 *s*

^&^OH: 11.36 *s*

^#^OH: 11.80 *s*

^$^OH: 11.36 *s*, 11.50* s*

**Table 6:** ^1^H-NMR spectroscopic data of compounds **29–36** (*δ* in ppm,* J* in Hz)

H	**29**^a#^ [44] 500 MHz	**30**^e$^ [45] 400 MHz	**31**^a%^ [46] 200 MHz	**32**^d&^ [46] 200 MHz	**33**^c^ [46] 200 MHz	**34**^e^ [46] 200 MHz	**35**^c^ [42] 200 MHz	**36**^e^ [33] 400 MHz
2	7.64 *s*	7.47 *s*	7.51 *s*	7.89 *s*	7.83 *s*	7.78 *s*	7.90 *s*	7.31 *s*
5	9.11 *d* (8.4)	8.29 *d* (1.4)	8.30 *d* (1.4)	8.78 *d* (9.3)	8.84 *d* (9.2)	8.16 *d* (2.4)	9.11 *m*	8.64 *d* (9.1)
6	7.90 *t* (7.8)			7.31 *d* (9.3)	7.32 *d* (9.2)		7.63 *m*	7.12 *d* (9.1)
7	7.80 *t* (7.6)	6.93 *d* (1.4)	6.92 *d* (1.4)			6.70 *d* (2.4)	7.63 *m*	
8	8.24 *d* (7.8)						7.87 *m*	
9	8.52 *s*	7.69 *d* (9.4)	7.77 *d* (9.4)	7.96 *d* (9.7)	8.05 *d* (9.8)	7.96 *d* (9.4)	7.71 *d* (9.6)	7.78 *d* (9.5)
10		8.77 *d* (9.4)	8.80 *d* (9.4)	8.96 *d* (9.7)	8.82 *d* (9.8)	8.50 *d* (9.4)	8.85 *d* (9.6)	8.27 *d* (9.5)
O-CH_2_-O	6.52 *t* (4.2)	6.25 *s* 6.19 *s*	6.30 *s* 6.24 *s*	6.41 *s*	6.32 *s*	6.31 *s*	6.35 *s*	6.20 *s*
OCH_3_		3.96 *s*	3.97 *s*	3.95 *s*	3.99 *s, *4.01 *s*, 4.03 *s*	4.00 *s*		3.92 *s*
CO_2_Me							4.00 *s*	
CONH	8.97 *br s*							

^a^DMSO-*d*_6_

^c^CDCl_3_

^d^(CD_3_)_2_CO

^e^CD_3_OD

^^^OH: 10.44 *s*

^#^Ala: 4.36 *s*, 1.42 *d* (7.3)

^$^OH: 5.38 *br s*, 4.99–5.19 *m*, 4.64 *m*, Glc: 5.11 *d* (6.0), 3.22–3.73 *m*

^%^OH: 5.39 *br s*, 4.90–5.20 *m*, 4.64 *t* (5.0), Glc: 5.08 *d* (5.6), 3.22–3.76 *m*

^&^OH: 8.61 *br s*

**Table 7:** ^13^C-NMR spectroscopic data of compounds **13**, **15**–**19**, **23**–**29** (*δ* in ppm)

C	**13**^a#^ [37] Unkown	**15**^a^ [38] 126 MHz	**16**^a^ [38] Unknown	**17**^a^ [39] 126 MHz	**18**^a^ [39] 126 MHz	**19**^a^ [39] 126 MHz	**23**^a^ [43] 100 MHz	**24**^a^ [17] 125 MHz	**25**^a^ [17] 125 MHz	**26**^a^ [17] 125 MHz	**27**^a^ [17] 125 MHz	**28**^c^ [24] 125 MHz	**29**^a$^ [44] 125 MHz
1	123.5	–^f^	124.1	–^f^	–^f^	124.2	118.0	124.6	124.3	123.6	124.3	124.6	128.1
2	112.1	111.6	110.8	114.2	111.5	111.0	112.6	113.8	112.6	112.1	113.9	109.9	111.8
3	145.1	145.3	145.8	145.6	145.3	145.9*	145.7	147.3	146.3	146.3	146.4	148.1	146.9
4	146.7	–^f^	146.3	142.5	143.9	147.0*	145.7	146.8	146.8	147.5	146.8	149.1	146.1
4a	116.3	–^f^	117.8	–^f^	116.4	117.9	117.0	117.3	117.6	118.3	117.4	119.4	117.8
4b	131.0	131.0	132.7	128.9	131.7	131.7	132.1	130.2	133.2	136.9	135.0	130.5	129.3
5	111.4	111.1	121.9	126.4	103.6	121.6*	104.1	127.8	112.0	129.8	115.6	104.1	127.0
6	157.9	159.2	122.8	129.3	160.3	122.9*	158.4	126.1	160.7	110.3	150.8	159.8	130.7
7	95.3	118.2	148.4	127.8	99.2	149.0	100.0	128.0	119.6	159.4	106.9	98.9	129.0
8	135.4	131.7	148.9	129.8	157.6	149.0	161.5	129.8	131.8	116.4	152.4	157.6	130.7
8a	122.5	122.6	122.8	129.0	112.2	122.0	112.3	135.5	122.4	126.3	120.3	111.9	129.3
9	130.3	–^f^	121.5	122.5	117.4	119.2	112.6	121.6	127.2	124.8	121.8	26.5	125.8
10	142.6	–^f^	142.9	148.6	145.1	142.9	145.7	146.0	144.3	145.6	146.3	78.8	145.4
10a	117.6	–^f^	115.5	116.8	–^f^	115.6	117.0	118.3	118.3	118.4	118.6	126.2	116.5
C = O	169.9	–^f^	–^f^	–^f^	–^f^	168.2	168.9	169.6	168.8	168.9	169.8	166.6	167.4
O-CH_2_-O	101.7	101.9	102.6	101.9	101.7	102.7	102.7	103.1	103.7	103.2	103.8	102.4	103.4
OCH_3_			60.9		56.0	61.0	56.6						

^a^DMSO-*d*_6_

^c^CDCl_3_

^f^data not shown [24]

^#^Glc: 100.6, 73.0, 77.3, 69.5, 76.6, 60.5

^$^Ala: 174.5, 48.4, 17.7

^*^interchangeable

**Table 8:** ^1^H- and ^13^C-NMR spectroscopic data of compounds **37**, **39–43** (*δ* in ppm, *J* in Hz)

H/C	**37**^a@^ [47] 400 MHz	**37**^a^ [47] 100 MHz		**39**^a^ [49] 600 MHz	**39**^a^ [49] 150 MHz		**40**^a^ [18] 500 MHz	**40**^a^ [18] 125 MHz	**41**^a^ [18] 500 MHz	**41**^a^ [18] 125 MHz	**42**^a^ [51] 60 MHz	**42**^d^ [50] 200 MHz	**42**^a^ [19] 100 MHz	**43**^c^ [51] 60 MHz
1		122.3				129.5		128.0		128.8			119.5	
2	7.88 *s*	112.1	7.78 *s*			108.9	7.45 *s*	108.5	7.41 *s*	108.3	7.89 *s*	7.98 *s*	111.7	7.74 *s*
3		144.6				143.9		143.9		146.1			144.3	
4		146.7				144.2		146.1		146.7			145.6	
4a		115.8				115.6		117.1		115.5			115.5	
4b		128.1				126.6		128.0		128.8			128.4	
5	9.05 *m*	126.8	9.01 *m*			126.7	8.78 *d* (8.3)	119.6	7.16 *d* (8.8)	116.1	8.65 *d* (8.0)	8.73 *d* (8.6)	118.7	8.70 *d* (8.2)
6	7.68 *m*	127.0	7.66 *m*			127.3	7.77 *t* (8.3)	128.4	7.36 *d* (8.75)	111.9	7.62 *t* (7.2)	7.61 *dd* (8.6, 8.2)	127.1	7.58 *t* (8.1)
7	7.68 *m*	127.7	7.66 *m*			127.3	7.44 *d* (6.8)	109.0		147.0	7.20 *d* (7.8)	7.21 *d* (8.2)	107.3	7.01 *d* (7.5)
8	7.97 *m*	128.1	7.96 *m*			127.9		153.9		149.9			154.7	
8a		131.4				131.5		115.3		119.8			121.8	
9	7.79 *d* (9.6)	127.0	7.70 *d* (9.0)			125.5		161.5		161.9	8.06 *d* (9.9)	8.16 *d* (9.8)	119.1	8.20 *d* (9.8)
10	8.80 *d* (9.6)	124.1	8.17 *d* (9.0)			124.3		145.8		144.7	8.81 *d* (9.8)	8.97 *d* (9.8)	123.9	8.80 *d* (9.7)
10a		127.7				125.2		118.6		118.6			128.0	
C = O		168.4				170.1		171.6		172.0			168.4	
O-CH_2_-O	6.44 *s*	102.5	6.39 *s*			101.9	6.42 *s*	102.1	6.27 *s*	101.6	6.42 *s*	6.44 *s*	102.5	6.28 *s*
OCH_3_							4.18 *s*	56.3	4.09 *s*	57.1	4.01 *s*	4.05 *s*	55.7	4.01 *s*
CO_2_Me														3.97 *s*
OCHO							8.22 *s*	161.5	8.20 *s*	162.0				

^a^DMSO-*d*_6_

^c^CDCl_3_

^d^(CD_3_)_2_CO

^@^OH: 13.13 *br s*

**Table 9:** ^1^H- and ^13^C-NMR spectroscopic data of compounds **44**–**49** (*δ* in ppm,* J* in Hz, CDCl_3_)

H/C	**44** [52] 400 MHz	**44** [52] 100 MHz	**45** [52] 400 MHz	**46** [52] 400 MHz	**46** [52] 100 MHz	**47** [52] 400 MHz	**48** [53] 400 MHz	**48** [53] 100 MHz	**49** [53] 400 MHz	**49** [53] 100 MHz
1		123.2			123.3			124.1		124.0
2	7.87 *s*	112.7	7.73 *s*	7.70 *s*	112.8	7.70 *s*	7.72 *s*	112.7	7.76 *s*	112.8
3		145.8			145.9			145.9		145.9
4		146.6			146.6			146.3		146.4
4a		118.3			118.4			118.4		118.4
4b		130.8			130.8			130.7		130.8
5	8.58 *d* (8.8)	118.9	9.13 *d* (8.4)	8.68 *dd* (8.5, 3.6)	119.1	9.13 *d* (8.4)	8.64 *d* (8.0)	119.1	8.68 *d* (8.2)	119.1
6	7.64 *t* (8.8)	130.9	7.79 *t* (8.4)	7.71 *t* (8.5)	131.0	7.81 *td* (8.4, 2.4)	7.68 *t* (8.0)	130.7	7.70 *t* (8.2)	130.8
7	7.04 *d* (8.8)	107.8	7.70 *t* (8.4)	7.10 *dd* (8.5, 3.6)	107.9	7.71 *td* (8.4, 2.4)	7.07 *d* (8.0)	107.8	7.10 *d* (8.2)	107.8
8		156.7	8.00 *d* (8.4)		156.9	7.99 *dd* (8.4, 2.4)		156.8		156.8
8a		119.9			120.1			120.1		120.2
9	8.79 *s*	121.2	8.35 *s*	8.84 *s*	121.3	8.34 *s*	8.79 *s*	120.8	8.81 *s*	120.9
10		145.5			145.6			145.7		145.9
10a		118.1			118.2			118.3		118.4
O-CH_2_-O	6.34 *s*	102.4	6.40 *s*	6.36 *s*	102.4	6.40 *s*	6.35 *d* (1.2) 6.36 *d* (1.2)	102.3	6.36 *d* (1.5) 6.37 *d* (1.5)	102.3
CO_2_H		166.0			165.8			166.3		166.2
OCH_3_	4.03 *s*	55.9		4.06 *s*	55.9		4.05 *s*	55.9	4.05 *s*	55.9
1′	10.25 *s*	191.2	10.25 *s*	9.51 *s*	195.9	9.52 *s*	5.81 *dd* (17.6, 10.8)	145.5	5.86 *dd* (17.5, 10.8)	145.6
2′		141.7			145.8		4.79 *d* (10.8) 4.98 *d* (17.6)	111.9	5.00 *d* (17.5) 4.94 *d* (10.8)	111.9
3′	6.08 *d* (11.2)	144.4	6.08 *d* (10.8)	6.36 *d* (10.6)	148.9	6.36 *d* (9.6)	6.19 *s*, 6.24 *s*	137.4	6.23 *s*	137.3
4′	6.37 *dd* (11.2, 4.6)	67.6	6.37 *m*	5.70 *td* (10.6, 3.6)	71.6	5.72 *td* (9.6, 3.4)		150.6		150.5
5′	2.72 *br d* (12.2) 2.39 *br d* (12.2)	44.9	2.73 *br d* (11.4) 2.40 *br d* (11.4)	2.80 *dd* (10.6, 3.6) 2.43 *t* (10.6)	43.6	2.81 *dd* (12.8, 3.4) 2.43 *dd* (12.8, 9.6)	3.42 *s*	43.5	3.43 *s*	43.4
6′		128.5			129.1			133.4		133.6
7′	5.04 *br d* (11.2)	130.6	5.05 *br d* (11.2)	5.10 *t* (7.0)	129.5	5.11 *t* (7.6)	5.77 *m*	126.0	5.71 *m*	125.7
8′	2.30 *br t* (11.2) 1.98 *br t* (11.2)	25.0	2.32 *br t* (11.2) 2.00 *br t* (11.2)	2.14 *t* (7.0)	24.9	2.15 *t* (7.6)	2.11 *br s*	22.8	2.09 *m*	22.9
9′	2.02 *m*, 2.20 *m*	39.6	2.03 *m*, 2.18 *m*	2.05 *m*	38.4	2.06 *m*	1.36 *m*	27.9	1.42 *m*	28.0
10′		134.9			133.6			38.3		38.3
11′	4.81 *br d* (8.4)	125.2	4.82 *br d* (8.4)	4.85 *t* (7.8)	125.5	4.85 *t* (7.6)	2.33 *dd* (14.4, 8.0) 1.95 *br d* (14.4)	42.3	2.30 *dd* (14.0, 5.3) 2.09 *m*	42.5
12′	2.07 *m*, 2.42 *m*	25.9	2.06 *m*, 2.42 *m*	2.21 *m*, 2.33 *m*	25.1	2.22 *m*, 2.32 *m*	5.17 *m*	71.1	5.09 *q* (6.5)	71.7
13′	2.84 *dt* (13.0, 4.0) 1.80 *td* (13.0, 4.0)	31.7	2.85 *br d* (12.4) 1.80 *br t* (12.4)	2.63 *dd* (9.8, 5.1) 2.40 *t* (9.8)	25.6	2.64 *dd* (9.7, 4.9) 2.40 *t* (9.7)	1.38 *d* (6.0)	20.1	1.34 *d* (6.5)	19.7
14′	1.61 *s*	15.9	1.61 *s*	1.68 *s*	18.6	1.69 *s*	9.63 *s*	194.2	9.66 *s*	194.2
15′	1.34 *s*	14.9	1.34 *s*	1.40 *s*	15.4	1.41 *s*	0.71 *s*	25.9	0.75 *s*	25.4

^f^data not observed [39]

**Table 10:** ^1^H- and ^13^C-NMR spectroscopic data of compounds **50**–**54** (*δ* in ppm,* J* in Hz, CDCl_3_)

H	**50** [53] 400 MHz	**51** [53] 400 MHz	**52**^#^ [54] 400 MHz	**52** [54] 100 MHz	**53** [39] 500 MHz	**53** [39] 126 MHz	**54** [39] 500 MHz	**54** [39] 126 MHz
1				125.0		–^f^		–
2	7.73 *s*	7.77 *s*	7.59 *s*	112.2	7.75 *s*	112.7	7.76 *s*	112.8
3				146.3		143.1		–^f^
4				146.8		147.5		146.8
4a				118.4		–^f^		–^f^
4b				130.8		131.0		–^f^
5	9.12 *d* (8.0)	9.13 *d* (8.3)	8.61 *d* (8.0)	119.1	8.72 *d* (7.5)	119.2	9.17 *d* (8.0)	127.4
6	7.79 *t* (8.0)	7.79 *t* (8.3)	7.64 *t* (8.0)	130.9	7.74 *dd* (7.5, 8.0)	131.0	7.83 *t* (8.0)	130.5
7	7.70 *t* (8.0)	7.73 *t* (8.3)	7.04 *d* (8.0)	107.9	7.13 *d* (8.0)	108.0	7.74 *t* (8.0)	–^f^
8	7.97 *d* (8.0)	7.98 *d* (8.3)		156.9		156.9	8.01 *d* (7.5)	130.2
8a				120.1		120.2		128.5
9	8.31 *s*	8.33 *s*	8.73 *s*	120.9	8.86 *s*	121.2	8.36 *s*	126.5
10				145.8		145.9		–^f^
10a				118.2		–^f^		118.3
O-CH_2_-O	6.38 *s*, 6.40 *s*	6.39 *d* (1.4), 6.40 *d* (1.4)	6.30 *s*	102.4	6.39 *s*	102.4	6.42 *s*	103.0
CO_2_H				167.3		167.2		167.5
OCH_3_			3.98 *s*	55.9	4.08 *s*	56.2		
1′	5.80 *dd* (17.6, 11.2)	5.85 *dd* (17.6, 10.8)		42.0	0.76 *dt* (13.0, 3.0) 1.80 *br d* (13.0)	40.3	0.76 *m* 1.80 *m*	40.4
2′	4.99 *d* (17.6) 4.79 *d* (11.2)	5.00 *dd* (17.6, 0.8) 4.91 *dd* (10.8, 0.8)		18.3	1.42 *m*	18.6	1.40 *m*	–^f^
3′	6.25 *s*, 6.20 *s*	6.24 *s*		42.0	1.15 *dt* (13.5, 4.0), 1.64 *m*	41.9	1.15 *m*, 1.64 *m*	42.0
4′				33.2		33.3		–^f^
5′	3.43 *s*	3.42 *s*		56.1	0.80 *m*	56.0	0.80 *m*	56.1
6′				20.4	1.56 *m*	20.5	1.56 *m*	–^f^
7′	5.78 *m*	5.72 *m*	2.02 *d* (12.0)	38.0	1.40 *m*, 1.52 *m*	42.1	1.38 *m*	42.0
8′	2.12 *br s*	2.10 *m*		44.5		44.9		–^f^
9′	1.36 *m*	1.44 *m*		56.5	1.06 *br d* (7.5)	56.7	1.07 *d* (7.4)	56.6
10′				39.4		39.4		–^f^
11′	1.95 *br d* (15.0) 2.33 *dd* (15.0, 8.8)	2.10 *m* 2.32 *dd* (14.2, 6.4)		18.5	1.62 *m*	18.3	1.62 *m*	–^f^
12′	5.17 *q* (6.5)	5.10 *q* (6.4)		26.2	1.62 *m*	26.3	1.62 *m*	27.1
13′	1.38 *d* (6.4)	1.35 *d* (6.4)	2.63 *br s*	43.4	2.12 *br s*	46.3	2.13 *br s*	46.5
14′	9.64 *s*	9.67 *s*		40.3	1.66 *m*, 1.98 *d* (11.5)	37.2	1.66 *m*, 1.99 *br d* (11.7)	37.5
15′	0.66 *s*	0.72 *s*	1.78 *d* (16.0) 1.91 *d* (16.0)	50.9	1.54 *br d* (14.5) 1.72 *dd* (14.5, 1.8)	53.3	1.54* m* 1.72 *m*	53.4
16′				98.1		80.1		80.0
17′			3.99 *dd* (11.5, 4.0) 4.33 *dd* (11.5, 4.0)	63.5	4.50 *d* (11.5) 4.54 *d* (11.5)	69.6	4.51 *d* (11.5) 4.54 *d* (11.5)	70.2
18′			0.78 *s*	33.5	0.87 *s*	33.6	0.87 *s*	33.6
19′			0.74 *s*	21.5	0.82 *s*	21.5	0.82 *s*	22.0
20′			0.97 *s*	17.1	1.04 *s*	17.8	1.04 *s*	18.0

^#^OH: 3.25 *br t* (4.0)

**Fig. 4:**
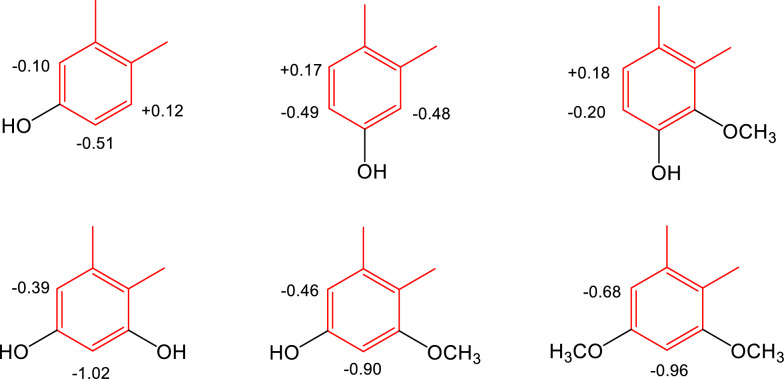
Substituent effects on C-ring of 3,4-methylenedioxyaristolochic acids

Aristolochic acids all have structures of 10-nitrophenanthrenecarboxylic acid. The majority of AAs have a 3,4-methylenedioxy group (phenanthro[3,4-*d*]-1,3-dioxole-10-nitro-1-carboxylic acids). In the IR spectra, the presence of the nitro group is indicated at *ν*_max_ *ca.* 1525 and 1346 cm^-1^, whereas the carbonyl group absorbs in the range 1710-1600 cm^-1^ [38]. At C-6, C-7, or C-8, the C ring of the phenanthrene skeleton is commonly substituted with a hydroxyl or a methoxy group, resulting in monooxygenated, deoxygenated, or trioxygenated phenanthrenecarboxylic acids. To confirm the locations of the hydroxyl or methoxy substituents of the C ring, NOESY experiments can be conducted [36]. In some examples, the C-ring is unsubstituted. Taken together, the following characteristics are visible in the ^1^H-NMR spectra of most AAs: the singlet signals of aromatic H-2 and H-9 at *δ*_H_ *ca* 7.70-7.80 and 8.20-8.70, respectively, and the proton spin-spin splitting of aromatic H-5 and H-8 in dependency of the aromatic substitution patterns of H-6, H-7, and H-8. C-5 is unsubstituted in all instances, and the proton signal for H-5 is often shifted downfield at *δ*_H_ *ca.* 8.20-8.70 because of the shielding effect of the phenanthrene A ring. The methylenedioxy group is indicated by a two-proton singlet signal at *δ*_H_ *ca*. 6.40, *δ*_C_ *ca.* 102-104. The common derivatives of AAs are their methyl esters [16, 31–33, 35, 40, 42], which may be inferred from an additional methoxy signal at *δ*_H_ *ca.* 4.00 in their ^1^H-NMR spectra. Comparisons of chemical shifts of the phenanthrene C-ring of 3,4-methylenedioxyaristolochic acids can lead to some remarks about substituent effects. Glucosylation at C-6 of AA I [17] (compound **1**, Table 3) causes upfield shifts of H-5 (-0.26 ppm) and H-7 (-0.20 ppm) in DMSO-*d*_6_. The shielding effects observed for **2** are H-5 (-0.16 ppm) and H-7 (-0.34 ppm). Methoxy and hydroxy groups are common substituents of the C-rings of AAs isolated from *Aristolochia* plants. 6-Oxy, 7-oxy, 6,8-dioxy, and 7,8-dioxy are substitution patterns observed in the current review, and the shielding (upfield shift) or deshielding effect (downfield shift) compared with AAII (**24**) in DMSO-*d*_6_ are summarized in Fig. 4. Occasionally, sodium aristolochates are isolated [38, 41]. Their salt form is indicated by the carboxyl group appears at *ν*_max_ *ca.* 1540-1580 cm^-1^ in the IR spectra [38]. In comparison between ^1^H- and ^13^C-NMR spectroscopic data of the free AAs and their sodium salts, the chemical shifts the carboxyl groups are at *δ*_C_ *ca.* 168 and C-1 at *δ*_C_ *ca.* 121-124, whereas H-9 of the sodium salts shifts upfield to *δ*_H_ *ca*. 8.15-8.35 (acids: *δ*_H_ *ca*. 8.45-8.60) [28, 29]. Treatment of the salt form with 5% HCl and the solution is purified on a Sephadex LH-20 column eluted successively with H_2_O and MeOH affords sodium chloride, which is determined by atomic absorption spectrometry [33, 38, 41]. The MeOH solution affords free acids, whose carbonyl group appears at *ν*_max_ *ca.* 1660-1710 cm^-1^ in the IR spectra [38]. Compared to those of the free acids H-9 of the sodium salts of AAs appeared slightly upfield at *δ*_H_ *ca* 8.15-8.35 [38].

**Fig. 5:**
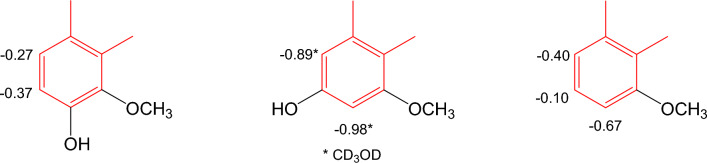
Substituent effects on C-ring of 3,4-methylenedioxyaristolic acids

Aristolochic acids can be converted into denitro compounds by reduction, both chemically [20, 50] and biologically [19], e.g., aristolochic acid I is converted to aristolic acid. Similar to the nitro compounds, the occurrence of denitroaristolochic acids as their methyl esters [34] or sodium salts [45] is frequently found in *Aristolochia* herbs. In one publication [47], primary amide derivatives of aristolic acids were isolated; the amide group was identified by an IR absorption band for a NH_2_ group at *ν*_max_ 3182 cm^-1^ and a carbon-13 signal at *δ*_C_ 170.1. Free carboxylic acids are identified by an IR absorption band for the carboxyl group at *ν*_max_ 3000 and 1670 cm^-1^ and a carbon-13 signal at *δ*_C_ *ca.* 168.0 [46]. The presence of the carboxyl group can be detected by D_2_O exchange. Al-Barham et al. reported the isolation of a secondary amide of aristolochic acid (**29**) [44], which has an IR absorption band for the NH group at *ν*_max_ 3466 cm^-1^ and an amide carbon-13 signal at *δ*_C_ 174.5. In addition, the NH signal in the ^1^H-NMR spectrum appears at *δ*_C_ 8.97 (*br s*). In accordance with the structural change to phenanthrene-1-carboxylic acids, the NMR spectra of the denitro derivatives show signals of a *cis*-configured C-9/C-10 double bond at *δ*_H_ *ca.* 8.80 and 7.70 (*J* ~ 9-10 Hz) and *δ*_C_ *ca*. 124-127. Glucosylation at C-6 of aristolic acid (**42**) (compound **31**, Table 6) causes upfield shifts of H-5 (-0.35 ppm) and H-7 (-0.28 ppm) in DMSO-*d*_6_. The shielding or deshielding effects of 8-oxy, 7,8-dioxy, and 6,8-dioxy substituents in DMSO-*d*_6_ compared with aristolic acid (**42**) are summarized in Fig. 5. Two first formyloxy derivatives of aristolic acids are reported in [18] with C-9 and C-10 substituted with a hydroxyl group and a formyloxy group, respectively. Consequently, the olefinic carbon/proton signals are not observed, instead, the formyloxy group resonances at *δ*_H_ *ca.* 8.20 and at *δ*_C_ *ca*. 162. Since the compounds occurred as their sodium salts, the cation portion was confirmed by ICP-MS.

Species of *Aristolochia* have been reported to contain elemane, caryophyllane, and humulane [12]. Mandolin R, mandolin L, mandolin M, and arystophyllene [12], which were previously isolated from the root and stem of *A. heterophylla*, may be related precursors for the sesquiterpene alcohol moieties of aristolochic acid esters from *Aristolochia* species, however, more research is needed to identify  the direct precursor compounds. *ent*-Kaurane diterpenoids are widely isolated from *Aristolochia* species, including *A. elegans* [12]. Epoxide ring-opening of the precursor 16,17-epoxy-*ent*-kaurane or by an esterification of AAs and *ent*-kaurane-16,17-diol may produce aristolin (**52**), aristoloins I (**53**) and II (**54**). These terpeno-aristolochic acid hybrid compounds are identified by ^13^C-NMR, which revealed 14 carbons for the core phenanthrene moiety, a carboxyl group, and 15 or 20 carbons for the sesqui- or diterpene moiety, respectively. Compounds **52-54** have been included as *Aristolochia* diterpenes in the review [13]. The HMBC correlations between the *α*-proton of the terpene alcohol moiety and the carbonyl group of AAs simplify the identification of the ester linkage. The ester itself exhibits an IR absorption band at *ν*_max_ 1710 cm^-1^. However, when the ester group is located at tertiary C-16 of the *ent*-kaurane-16*β*,17-diol [12], the upfield shifts of kaurane C-13 and C-17 (*δ*_C_ *ca.* 43.4 and 63.5 in CDCl_3_, respectively) are indicative for the ester location. Kaurane C-16 is anticipated to undergo a downfield shift, e.g, the carbon-13 chemical shift was at *δ*_C_ 98.1 for *ent*-kaurane C-16 of **52** [39]. Compounds **44-47** possess several chiral centers of the *ent*-elemane moiety. Their absolute stereostructures were determined by using the CD (Circular Dichroism) exciton chirality method [52]. The experimental positive and negative Cotton effects were compared with the chiral exciton couplings between the cyclohexene double bond and the unsaturated aldehyde chromophore (at *ca.* 220 nm) and between the ring double bond and the 3,4-methylenedioxybenzoate chromophore (at *ca.* 260 nm). The ^13^C-NMR of compounds **45** and **47** could not be achieved due to small amounts of the isolated samples [52].

## Conclusion

More aristolochic acid derivatives have been isolated and reported since the early assessment of a few reviews on NMR spectroscopic data of common *Aristolochia* phenenthrene derivatives [27–29]. This review provides an update list of NMR spectroscopic data of AAs isolated from *Aristolochia* herbs. The NMR characteristics of three classes of derivatives, AAs, denitroaristolochic acids, and esters of AAs, are also been briefly discussed. By comparing NMR data, the data would facilitate the identification of the bioactive and toxic AAs in *Arisolochia* herbs. In addition, the latest development in analytical and preparative separation of AAs in *Aristolochia* herbs appears for the first time in the current review.

## Data Availability

Not applicable.
